# Subjective Mouthfeel and Temperature Alterations in COVID-19 Patients Six to Ten Months After Diagnosis

**DOI:** 10.1007/s12078-022-09304-y

**Published:** 2022-11-12

**Authors:** Jip M. van Elst, Sanne Boesveldt, Arjan Vissink, Harriët Jager-Wittenaar, Anna K. L. Reyners, Jacco J. de Haan

**Affiliations:** 1 Department of Medical Oncology, University of Groningen, University Medical Center Groningen, Groningen, The Netherlands; 2 Division of Human Nutrition and Health, Wageningen University & Research, Wageningen, The Netherlands; 3 Department of Oral and Maxillofacial Surgery, University of Groningen, University Medical Center Groningen, Groningen, The Netherlands; 4 Research Group Healthy Ageing, Allied Health Care and Nursing, Hanze University of Applied Sciences, Groningen, The Netherlands

**Keywords:** COVID-19, Long COVID, Quality of life, Smell, Taste, Mouthfeel

## Abstract

**Introduction:**

The characteristics and impact of mouthfeel, temperature, smell, and taste alterations in patients with COVID-19 at a long term are yet not well known. In this study, these characteristics and their impact on daily life and quality of life (QoL) were assessed, six to ten months after infection, in patients with COVID-19 searching for peer support on Facebook.

**Methods:**

Between December 2020 and January 2021, members of two COVID-19 Facebook groups were invited to complete a questionnaire. Participants were asked to report their perception of mouthfeel, temperature, smell, and taste alterations and their impact.

**Results:**

The questionnaire was completed by 157/216 respondents (73%), with 92% being women. Alterations in mouthfeel, temperature, smell, and taste were reported by respectively 66, 40, 148, and 133 participants. The most frequently reported mouthfeel alterations were “a different feeling” and “dry mouth” in 38 and 30 participants, respectively. Preferences for food temperature were equally changed to “freezing”, “cool”, “room temperature”, “a bit warmer”, and “warmer”. An impact on daily life and QoL was reported by most patients with alterations in mouthfeel (91% and 79%), temperature (78% and 60%), smell (98% and 93%), and taste (93% and 88%), respectively.

**Conclusions:**

Patients with COVID-19 searching for peer support on Facebook experienced, next to smell and taste alterations, mouthfeel and temperature disturbances, six to ten months after infection. These alterations have an impact on daily life and QoL.

**Implications:**

Health professionals should, next to smell and taste alterations, be aware of mouthfeel and temperature alterations in patients with COVID-19.

**Supplementary Information:**

The online version contains supplementary material available at 10.1007/s12078-022-09304-y.

## Introduction

Alterations in smell and taste are common symptoms in the acute phase of coronavirus disease 2019 (COVID-19) (Lechien et al. 2020; Russell et al. 2020; Ohla et al. 2022). Smell alterations, including anosmia, hyposmia, and parosmia, are present in 29–67% of the patients during the acute phase of the COVID-19, irrespective of the presence of nasal congestion (Parma et al. 2020; Agyeman et al. 2020; Wu et al. 2022). Taste alterations, including ageusia, hypogeusia, and dysgeusia, have been observed in 16–60% of patients with COVID-19 (Agyeman et al. 2020; Wu et al. 2022; Hajikhani et al. 2020). Smell and taste are important in the perception and enjoyment of food and drinks. Mouthfeel and temperature are also important aspects in this context; however, mouthfeel and temperature alterations have not been thoroughly studied in patients with COVID-19. The term mouthfeel describes sensations, which include cooling-, burning-, or tingling-like sensations in the mouth, that are related to food and drinks (Simons et al. 2019). One study reported a loss of chemesthesis or changes in mouthfeel in 46% of patients with COVID-19 (Parma et al. 2020).

Smell and taste alterations can have a profound impact on daily life and quality of life (QoL). Daily life can be impaired by safety issues, e.g., the inability to detect rotten food, gas leaks, and smoke, or having difficulty with cooking. QoL can be decreased by worrying about safety issues as well as the inability to control personal hygiene. Both smell and taste alterations affect QoL by decreasing food liking and appetite, which can reduce dietary intake and result in involuntary weight loss (Miwa et al. 2001; Coelho et al. 2021). Furthermore, social dysfunction and a higher rate of depression and anxiety are present among patients with these alterations (Philpott and Boak 2014; Croy et al. 2014). The impact of mouthfeel alterations has been studied less than the impact of smell and taste alterations, but has impact on QoL too (Merkonidis et al. 2015; Souza et al. 2011).

Patients with COVID-19 can have symptoms for months (long COVID), reducing QoL and participation in society (Desai et al. 2022; Malik et al. 2022; Elkholi et al. 2021). Consequently, it is important to have insight in the course of smell and taste alterations and associated symptoms, as well as their impact on daily life and QoL. Whereas most patients recover from olfactory dysfunction within two weeks, in a subgroup of patients, olfactory dysfunction appears to be permanent (Jafar et al. 2021; Borsetto et al. 2020). Patients with persisting alterations can search for peer support on social media (Gavrila et al. 2019). The large number of patients with COVID-19 and the likely presence of COVID-19 worldwide in the following years stresses the urgency of analyzing the characteristics and impact of persisting mouthfeel, temperature, smell, and taste alterations in a subgroup of patients who experience these alterations and who actively look for peer support. Knowledge on these alterations and on the patient’s request for help could lead to more personalized guidance of patients with COVID-19 and their relatives by their physician and/or dietitian. Social media like Facebook (FB) are an attractive option to reach and study the characteristics of a large group of patients with COVID-19 with smell and taste alterations in peer support groups. However, using FB as a recruitment method may limit generalization of results due to selection bias.

This study is aimed at identifying the characteristics of mouthfeel and temperature, next to smell and taste alterations as well as their impact on daily life and QoL in a population of patients with COVID-19 six to ten months after diagnosis who search for peer support on FB.

## Materials and Methods

### Study Population

This exploratory study was conducted between December 2020 and January 2021 in two Dutch FB groups: “Corona patiënten met langdurige klachten (Nederland)” (translated: Corona patients with long term complaints (the Netherlands)) and “Reuk- en smaakverlies na COVID-19” (translated: Smell and taste loss after COVID-19) (Facebook 2020a; Facebook 2020b). On December 2, 2020, the FB group “Corona patiënten met langdurige klachten (Nederland)” had over 17,000 members and “Reuk- en smaakverlies na COVID-19” had over 4,000 members. In these groups, patients with COVID-19 tend to look for peer support. Therefore, the prevalence of reported smell and taste alterations will be higher than in the general COVID-19 population. This way, the characteristics of these reported alterations and mouthfeel and temperature alterations in this group of patients can be thoroughly studied, and the impact of reported mouthfeel and temperature alterations can be compared to the impact of smell and taste alterations.

As FB is a transient medium, a small response rate was expected (Gu et al. 2016; Fan and Yan 2010). An earlier study performed in a FB group of similar size obtained meaningful results from 345 participants, and it was expected that in this study, a similar sample size could be realized (Goërtz et al. 2020).

### Study Procedure

Participants were recruited with an opt-in system. Participants were included if they reported to have had a positive PCR test for the SARS-CoV-2 virus in six to ten months prior to completing the questionnaire, were 18 years or older, and understood the Dutch language. Participants were excluded if they used medication that is known to strongly affect smell or taste. At the early start of the COVID-19 pandemic, PCR testing was not regularly performed in the Netherlands and few patients knew for sure they had a COVID-19 infection. Therefore, a longer time-frame of six to ten months after COVID-19 infection was chosen to study long-term symptoms.

A message with a link to the study website was posted twice on the FB page, on December 2, 2020 and January 6, 2021, with a brief explanation of the study. The first post was a “regular” FB group post, which was replaced by other posts within a day due to the active community posting other messages. To recruit more participants, a second message was posted and pinned at the top of the page’s timeline. This second post was visible for two weeks.

REDCap (Research Electronic Data Capture, Version 10.0.23), an application to support data capture in a safe web-based environment, was used to construct a website and collect the data, hosted at the University Medical Center Groningen (UMCG) (Harris et al. 2009). This website contained detailed information about the study for potential participants. Once informed consent was obtained, participants were forwarded to the questionnaire. The collected data could not be traced back to individual participants. The medical ethics committee of the UMCG approved the study (METc 2020/604). The study was conducted according to the Dutch law and complied with the Declaration of Helsinki for Medical Research involving Human Subjects. The study was registered in the Netherlands Trial Register (NTR NL9238).

### Questionnaire

The questionnaire was based on previous questionnaires (Online Resource 1) (van Elst et al. 2022; de Haan et al. 2021; IJpma et al. 2017). The questionnaire contained five parts. The first and second parts were completed by all participants, the third and fourth parts were only completed by participants who reported changes in, respectively, smell and taste, and the fifth part was completed by participants who reported smell and/or taste alterations. In the first part, general questions regarding demographic characteristics, hospitalization due to COVID-19, and medication were asked. The second part of the questionnaire addressed mouthfeel, temperature, smell, and taste alterations. In the questionnaire, we operationalized mouthfeel defined as “sensations in the mouth that are related to food and drinks” (Simons et al. 2019). In the third and fourth parts, participants were asked for their alterations in smell and taste, respectively. In the last part of the questionnaire, participants answered one question on the need for help or guidance with smell and or taste alterations. It took about 15 minutes to complete the entire questionnaire.

### Statistical Analyses

Statistical analyses were conducted with IBM SPSS Statistics 23 (IBM Corp., Armonk, NY). Participants with missing data were deleted pairwise. Descriptive statistics were reported as number with percentage or median with interquartile range. Spearman’s rank correlation coefficient was used to determine the correlation between characteristics of the participants (age or the number of months after the positive test), the severity of reported smell and taste alterations, and impact on daily life and QoL. A correlation with an *r* value < 0.3 was considered poor, 0.3 to 0.5 as fair, 0.6 up to 0.8 as moderately strong, and > 0.8 as very strong (Chan 2003). The Friedman test was used to study the differences between the severity of reported alterations in salt, bitter, sweet, and sour taste. The Friedman test and post hoc Wilcoxon signed-rank test were used to investigate the differences between reported mouthfeel, temperature, smell, and taste alterations in impact on daily life and QoL. In all analyses, *p* < 0.05 was considered significant. The *p* value was adjusted using the Bonferroni correction in the post hoc Wilcoxon signed-rank test and was significant at *p* < 0.008.

## Results

### Study Population

**Fig. 1:**
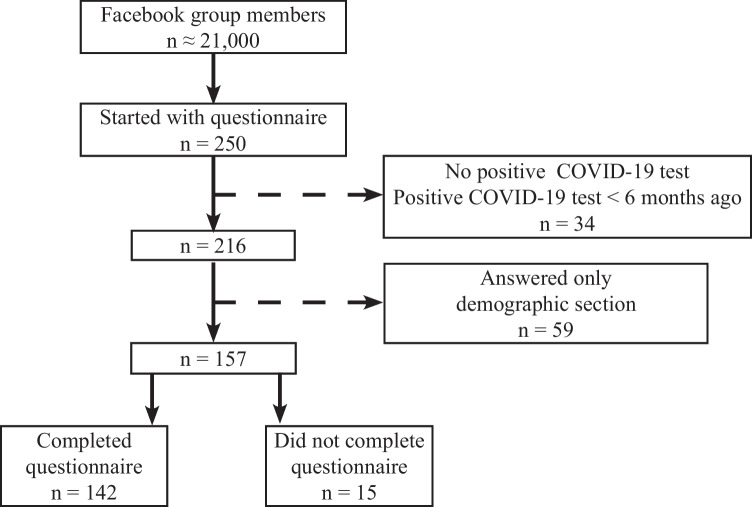
Inclusion and exclusion of participants (*n*) with COVID-19 infection

Of the ~ 21,000 FB group members, 250 individuals started completing the questionnaire (1%). Due to lack of a positive COVID test or a positive COVID test less than six months ago, 34 of the 250 individuals could not be included. Of the 216 individuals, 59 individuals only answered the demographic questions and were therefore not included. In total, 157 participants filled out questions regarding mouthfeel, temperature, smell, and taste alterations, of which 15 participants did not complete the full questionnaire (Fig. 1). Of the 157 participants, 92% were female and most had tested positive for COVID-19 in March 2020 (46%) or April 2020 (38%). The hospitalization rate of the participants was 10% (*n* = 16). The hospitalization group (intensive care (IC) and nursing ward) was too small to study differences between the hospitalized and non-hospitalized participants. Characteristics of the participants are described in Table 1.

**Table 1:** Baseline characteristics

Characteristics	Total (*n* = 157)	Patients previously hospitalized for COVID-19 (*n* = 16)	Non-hospitalized patients (*n* = 141)
Gender, *n* (%)			
*Male*	13 (8%)	2 (13%)	11 (8%)
Age (years), median [IQR]	47 [34–54]	51 [44–59]	46 [33–54]
Months since positive COVID-19 test, median [IQR]	9 [8–10]	9 [9–10]	9 [8–9]
Hospitalization, *n* (%)			
*IC*	3 (2%)	3 (19%)	n/a
*Nursing ward*	13 (8%)	13 (81%)	n/a
Hospitalization (days), median [IQR]	n/a	6 [4–13]	n/a

*n*, number; COVID-19, coronavirus disease 2019; IQR, interquartile range; IC, intensive care; n/a, not applicable

### Characteristics of Reported Mouthfeel, Temperature, Smell, and Taste Alterations

**Fig. 2:**
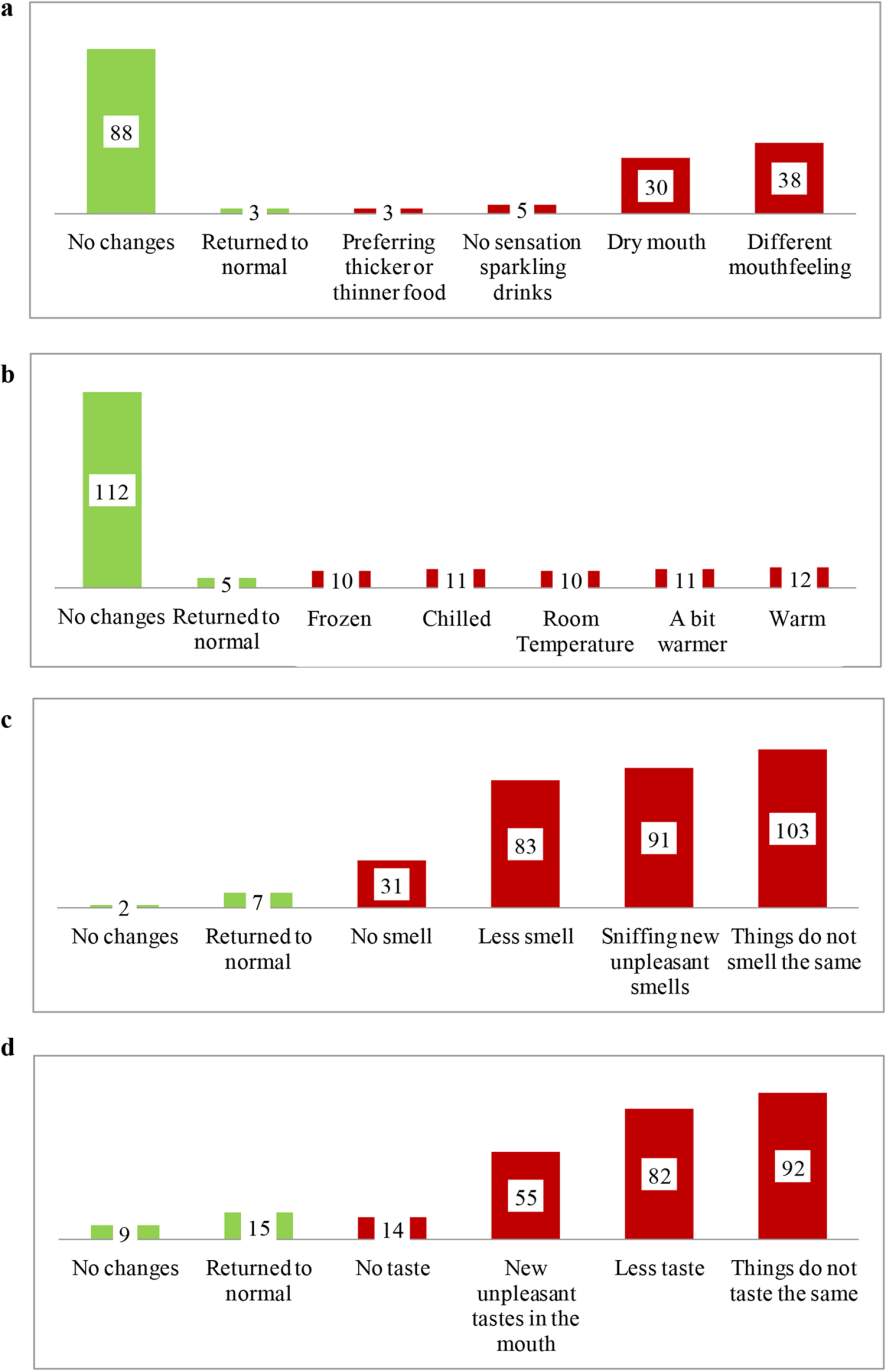
Self-reported type of alteration in **a** mouthfeel (*n* = 66), **b** temperature (*n* = 39), **c** smell (*n* = 145), and **d** taste (*n* = 131) in patients with COVID-19 (*n* = 157). Participants could indicate more than one type of alteration

An altered mouthfeel was experienced by 66/157 (42%) of the participants. These participants described mostly “a different feeling in the mouth” (*n* = 38/66, 58%) or “a dry mouth” (*n* = 30/66, 45%) (Fig. 2a). The preference for serving temperature of foods and drinks was altered in 40/157 (25%) of the participants. The preferences were equally changed from their normal preference to “freezing,” “cool,” “room temperature,” “a bit warmer,” and “warmer” (Fig. 2b).

**Fig. 3:**
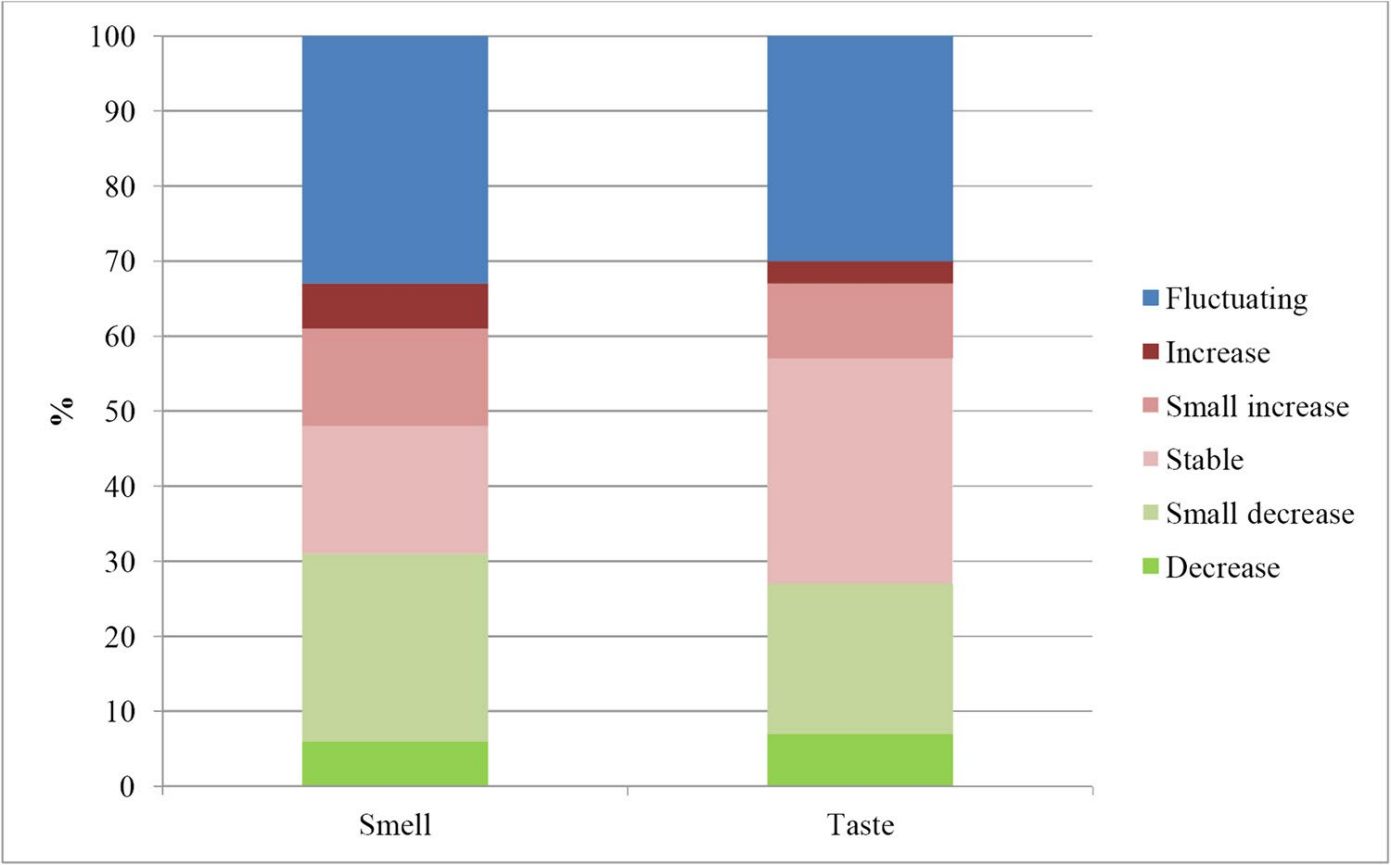
Proportion of recalled course of smell (*n* = 135) and taste (*n* = 116) alterations

Smell alterations, compared to prior to COVID-19, were reported by 148/157 participants. The types of changes described most often by participants with smell alterations were “things do not smell the same” (*n* = 103/148, 70%), “sniffing new unpleasant smells” (*n* = 91/148, 61%), and “less smell” (*n* = 83/148, 56%) (Fig. 2c). Of the participants with smell alterations, six did not answer further questions, and seven (5%) stated later that smell had not changed at all. Of the remaining 135 participants, 43 (32%) participants with smell alterations reported that they experienced their smell had altered a little, 55 (41%) that it changed quite a bit, and 37 (27%) that it changed very much. Most participants with smell alterations reported a fluctuating course of these alterations (*n* = 45/135, 33%) (Fig. 3). Scents that were reported to smell stronger included cleaning products, perfume, smells from the kitchen, onion, or sweat. Forty-three (27%) participants experienced no stronger smelling scents. A total of 24 (18%) participants experienced nasal congestion at the start of COVID-19, but not anymore. Permanent nasal congestion was described only by 20 (15%) participants with smell alterations.

Alterations in taste were described by 133/157 participants. Most participants experienced “that things did not taste the same” (*n* = 92/133, 69%), “less taste” (*n* = 82/133, 62%), and “new unpleasant tastes in the mouth” (*n* = 55/133, 41%) (Fig. 2d). Eight and nine participants, respectively, stopped filling in the questionnaire or stated no changes in taste at all. A little change was reported by 37/116 (32%), quite a bit change by 45/116 (39%), and very much change by 34/116 (29%) of the participants. The course of the reported taste alterations was mostly fluctuating or stable, both in 35/116 (30%) participants (Fig. 3).

**Fig. 4:**
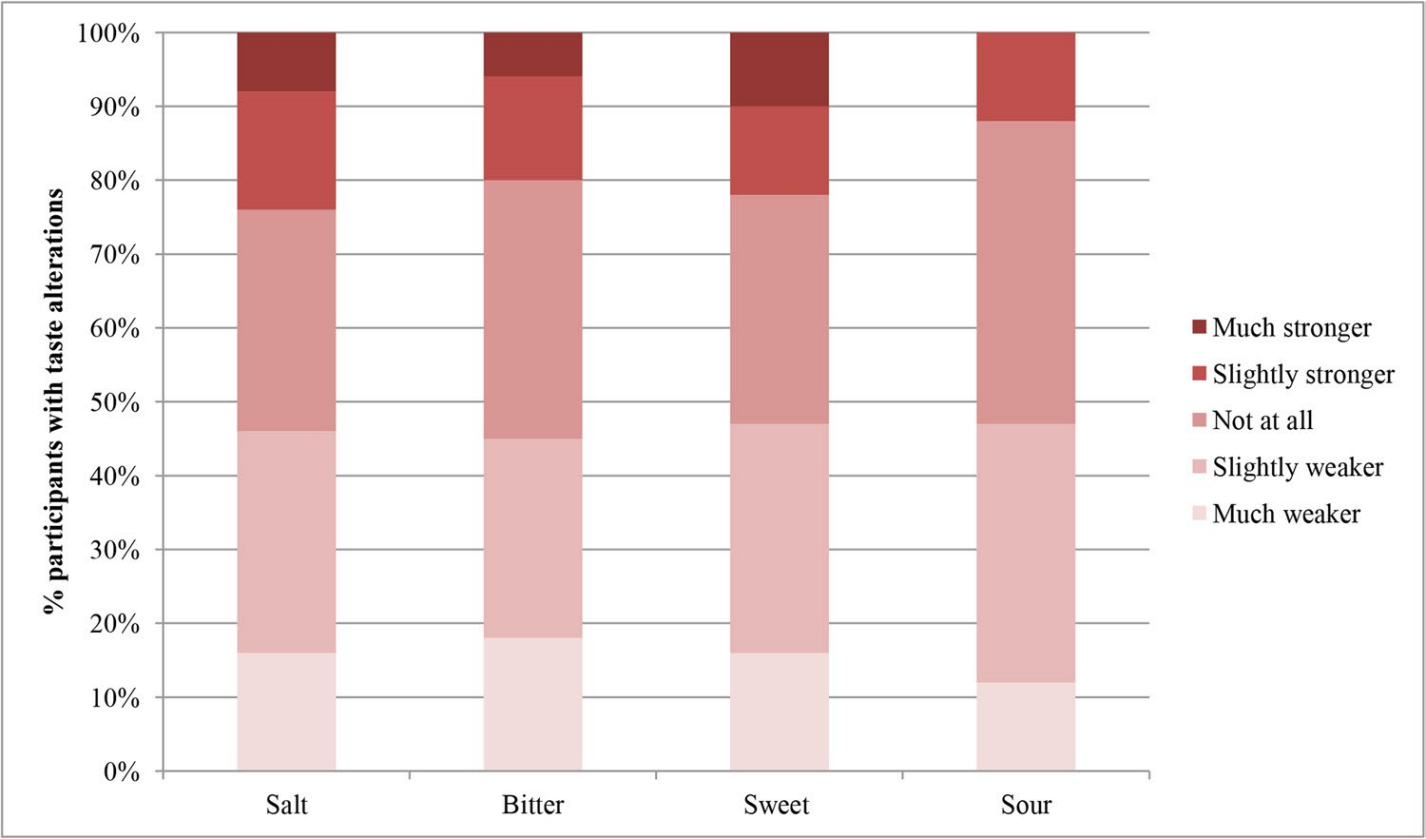
Proportion of reported alterations in salt, bitter, sweet, and sour taste in patients with COVID-19 (*n* = 113)

No difference between reported alterations in the basic tastes was observed (*p* = 0.794) (Fig. 4). A metallic taste was reported by 40/116 (34%) participants of whom 24 (21%) experienced the metal taste during meals and 16 (14%) in between meals. Of the participants with reported taste alterations, 60/116 (52%) did experience a continuous taste. The participants who experienced this continuous taste described this taste mostly as a “chemical” or “stale” taste.

### Impact on Daily Life and QoL

**Fig. 5:**
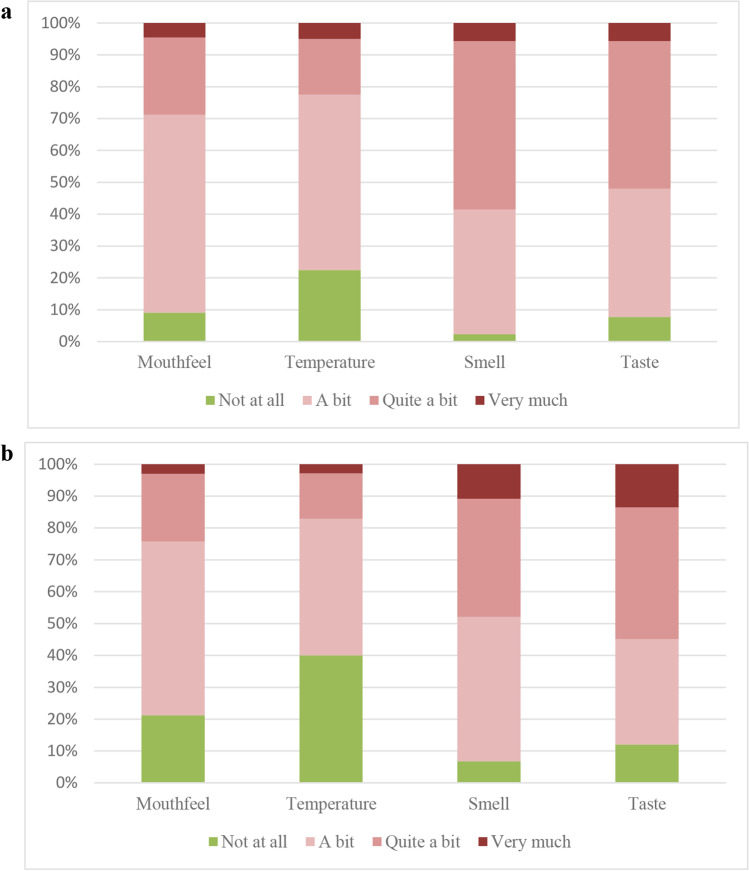
Impact of mouthfeel (*n* = 66), temperature (*n* = 40), smell (*n* = 148), and taste (*n* = 133) on **a** daily life and **b** QoL

The impact on daily life was not equally affected in the mouthfeel, temperature, smell, and taste alterations groups (*p* < 0.001) (Fig. 5a). Alterations in smell and taste were reported to have more impact on daily life (145/148 (98%) and 124/133 (93%) participants, respectively) than alterations in mouthfeel or temperature (60/66 (91%) and 31/40 (78%), respectively) (smell vs. mouthfeel, *Z* =  − 5.291, *p* < 0.001; smell vs. temperature, *Z* =  − 4.882, *p* < 0.001; taste vs. mouthfeel, *Z* =  − 4.468, *p* < 0.001; taste vs. temperature, *Z* =  − 4.422, *p* < 0.001). No differences were observed between the impact of smell versus taste alterations on daily life (*Z* =  − 2.101, *p* = 0.036).

QoL diminished in participants with altered mouthfeel, temperature, smell, and taste, respectively, in 52/66 (79%), 21/40 (60%), 138/148 (93%), and 117/133 (88%) participants (Fig. 5b). The QoL was not equally affected in these groups (*p* < 0.001). Alterations in smell and taste were reported to have more impact on QoL than alterations in mouthfeel or temperature (smell vs. mouthfeel, *Z* =  − 4.845, *p* < 0.001; smell vs. temp, *Z* =  − 4.518, *p* < 0.001; taste vs. mouthfeel, *Z* =  − 4.768, *p* < 0.001; taste vs. temperature, *Z* =  − 4.172, *p* = 0.001). No differences were found between the impact of smell versus taste alterations on QoL (*Z* =  − 0.290, *p* = 0.772).

Of the participants with an unpleasant or changed smell, 57/137 (42%) reported to do not anything to cope with the described changes in smell, and 49/137 (36%) reported to eat different food with bearable smells. To adapt to a diminished smell, 100/140 (71%) participants reported that they smelled scented products to see whether they smelled something, 94/140 (67%) participants described to ask others to smell if food was still good and 63/140 (45%) participants reported to pay more attention to personal hygiene. Changes in taste were mostly described to be counteracted by experimenting with stronger flavors (*n* = 51/116, 44%). However, 39/116 (34%) participants reported to do not anything to cope with the changes in taste. Whereas most participants undertook no specific actions to cope with changes in salt (*n* = 23/65, 35%), bitter (*n* = 32/52, 62%), sweet (*n* = 39/64, 61%), and sour (*n* = 27/50, 54%), changes in patients described they made most were using extra salt (*n* = 22/65, 34%) and avoiding bitter foods (*n* = 16/52, 31%).

Of the participants with reported smell or taste alterations, 101/130 (78%) would like to receive support. The support they described to wish most for was more information about smell or taste alterations (*n* = 67), an (experimental) drug treatment (*n* = 53), or advice from a dietitian (*n* = 32).

No correlations were found between age and time since the positive COVID-19 test and impact on daily life and QoL in reported mouthfeel, temperature, smell, and taste alterations. A fair correlation was found between the severity of reported smell and taste alterations (*r* = 0.457; *p* < 0.001). The severity of smell alterations had a fair correlation with the impact on daily life and QoL (*r* = 0.331; *p* < 0.001 and *r* = 0.335; *p* < 0.001, respectively). The correlation between severity of taste alterations and impact on daily life and QoL was fair too (*r* = 0.495; *p* < 0.001 and *r* = 0.456; *p* < 0.001, respectively).

## Discussion

The findings of this study indicate that a substantial group of patients with COVID-19 searching for peer support on FB experience disturbed mouthfeel and temperature six to ten months after infection, besides smell and taste alterations. We found large heterogeneity in the types (less/no/different smell or taste) of reported smell and taste alterations in patients with these changes. Interestingly, alterations did not differ between the basic tastes. All reported alterations had impact on daily life and QoL, although smell and taste alterations had more impact than mouthfeel and temperature alterations.

The heterogeneity of the symptoms and course of the smell and taste alterations since COVID-19 infection is in line with other studies, but this had not been studied specifically for mouthfeel and temperature alterations (Lechien et al. 2020; Parma et al. 2020; Burges Watson et al. 2021). Our study showed diminished QoL due to COVID-19-related smell and taste alterations, both on short and long term, which is in line with other studies (Lechien et al. 2020; Saniasiaya and Prepageran 2021). In a study with patients with COVID-19 from twelve European hospitals (both hospitalized and non-hospitalized), anosmia had the highest impact on QoL, compared to normosmia and hyposmia (Lechien et al. 2020). Our finding that reported mouthfeel and temperature alterations have less impact on daily life and QoL than smell and taste could be explained by the adaptation to temperature alterations being easier than adaptation to smell and taste alterations, for example, by serving colder or warmer food and drinks.

Many patients search for peer support on social media. Facebook is the largest social medium in the Netherlands (Gavrila et al. 2019; van der Veer et al. 2020). Therefore, peer support groups on Facebook are an attractive recruitment option to reach patients with COVID-19 and study the characteristics of a large group of patients with COVID-19 with smell and taste alterations. Advantages of FB recruitment for health-related research are reduction in costs and shorter periods of enrollment with more participants compared to conventional recruiting (Whitaker et al. 2017). However, the use of FB and other social media has its limitations. As it is a transient medium in which the algorithm does not show all messages, the response rate by FB users is usually low, as exemplified by 1.2% in our study (Gu et al. 2016; Fan et al. 2010; Sammut et al. 2021). An earlier study with patients with COVID-19 used a FB recruitment method that is comparable to the present study and had a similar response rate for confirmed patients with COVID-19 (Goërtz et al. 2020). Remarkably, both in our study and this previous study, more women, i.e., 92% and 85% respectively, were included. Women are overrepresented on FB and in FB studies and tend to report more symptoms than men, in general (Bardel et al. 2019; Gilmour et al. 2019).

A strength of this study is the inclusion of people mostly with a higher symptom burden, as FB groups for patients with COVID-19 and smell and taste alterations will attract these people. We could therefore study mouthfeel, temperature, smell, and taste alterations in a group with frequently reported occurrence of smell and taste alterations to better understand the problems they perceive. Therefore, the strength of this study lays in its contribution to the knowledge on mouthfeel and temperature changes and its impact on daily life and QoL, as well as providing insight in the type of smell and taste alterations and coping strategies. However, selection bias is method-inherent.

Participants recruited on FB are generally younger than participants who are recruited via traditional, non-social media approaches (Reagan et al. 2019). Older adults have the highest risk of a severe COVID-19 infection and experience most symptoms, but are underreported in this study (Luo et al. 2020). However, a younger population has the advantage of having less risk factors for affected smell and taste, like older age itself or comorbidities (Risso et al. 2020). Another limitation is that this study compared the difference in mouthfeel and temperature alterations before and after COVID-19 infection by asking participants specifically about these differences. However, the objective presence of mouthfeel and temperature alterations before COVID-19 infection in participants is not known. At last, the participants in this study likely had the SARS-CoV-2 alpha variant, as they were infected during the early phase of the pandemic. Furthermore, the participants were (yet) not vaccinated. Future studies should take detailed differences and recovery rates of mouthfeel, temperature, smell, and taste alterations between SARS-CoV-2 variants and vaccination-status into account (Boscolo-Rizzo et al. 2022; Vaira et al. 2022).

To conclude, patients with COVID-19 searching for peer support on FB experience mouthfeel and temperature alterations. These alterations, next to smell and taste alterations, have impact on daily life and QoL. Most patients did not change their behavior to cope with the reported alterations or avoided certain scents or foods, although others tried to adapt to this new situation by adjusting food and drinks. Whereas the impact on daily life and QoL of mouthfeel and temperature alterations is more modest than smell and taste alterations, it is an underreported problem which should receive more attention by health care professionals.

## Supplementary Information

Below is the link to the electronic supplementary material.

Supplementary file1 (PDF 268 KB)
