# Supplementary material for: Subjective Mouthfeel and Temperature Alterations in COVID-19 Patients Six to Ten Months After Diagnosis
Source: Chemosens Percept. 2022 Nov 12;15(2):165–74. doi: 10.1007/s12078-022-09304-y (PMC9660145; doi:10.1007/s12078-022-09304-y)
Supplement: Supplementary file 1 — Supplementary file1 (PDF 268 KB) [file 12078_2022_9304_MOESM1_ESM.pdf]

Supplementary material

Title: Subjective mouthfeel and temperature alterations in COVID-19 patients six to ten months after diagnosis

Chemosensory Perception

Authors: Jip M. van Elst, Sanne Boesveldt, Arjan Vissink, Harriët Jager-Wittenaar, Anna K.L. Reyners, Jacco J. de Haan*

*Corresponding author:

Affiliation: University of Groningen, University Medical Center Groningen, Department of Medical Oncology, Groningen, the Netherlands

E-mail: j.j.de.haan@umcg.nl

A. Questionnaire on sensory changes

Informed consent

Thank you for your interest in this study. ❑
Did you read the information above about this study and do you agree with this?

Patient characteristics

Q1a.	What is your age?
	**< 18? RESPONDENT IS FINISHED**

Q1b.	What is your gender?<br><br>**CIRCLE ONE CODE ONLY**	
	Male	1
	Female	2
	Other	3

Q1c.	In which month have you been positively tested for the Coronavirus?<br><br>**CIRCLE ONE CODE ONLY**		
	February 2020	1	**CONTINUE TO Q1d**
	March 2020	2	
	April 2020	3	
	May 2020	4	
	June 2020	5	
	July 2020	6	
	Later than July 2020	7	**RESPONDENT IS FINISHED**
	I don’t have a positive corona test	8	

Q1d.	Have you been admitted in the hospital due to your corona infection?<br><br>**OMCIRKEL SLECHTS ÉÉN ANTWOORD**	
Yes, on the Intensive Care	1	**CONTINUE TO Q1e**
Yes, on the nursing department	2	
No	3	**SKIP TO Q1f**

Q1e.	How many days have you been admitted in the hospital due to your corona infection?<br><br>**OPEN QUESTION**

Q1g.	Do you have other diseases for which you are treated with medication?<br><br>**OPEN QUESTION**

Taste, smell, texture and temperature changes

Q2a.	How has your **sense of smell** changed compared to before being positively tested for the corona virus, even if just a little?<br><br>**CIRCLE ONE CODE ONLY**		
	I do not perceive any smell at all	1	**CONTINUE TO Q2b**
	I perceive a difference in intensity of smells in general/lack of smell (smell acuity)	2	
	I don't perceive food smells in the same way I did before (smell alteration)	3	
	I detect new bad smells	4	
	I did have changes in smell, but not anymore	5	**SKIP TO Q3a**
	I do not perceive any change in smell perception	6	

Q2b.	**ONLY ASK THOSE WHO SELECTED 1-4 IN Q2a**<br>Thinking about the things that have changed your **sense of smell**, how much does it impact your daily life?<br><br>**CIRCLE ONE CODE ONLY**			
	Not at all	A little	Quite a bit	Very much
Smell	1	2	3	4

Q2c.	Thinking about the things that have changed your **sense of smell,** is your quality of life negatively affected because of these changes in taste?<br><br>**CIRCLE ONE CODE ONLY**			
	Not at all	A little	Quite a bit	Very much
Smell	1	2	3	4

Q3a.	How has your **sense of taste** changed compared to before being positively tested for the corona virus, even if just a little?<br><br>**CIRCLE ALL THAT APPLY**	
I do not perceive any taste anymore	1	**CONTINUE TO Q3b**
I perceive a difference in intensity of tastes in general (lack of taste)	2	
I don't perceive certain tastes the same way I did before (taste alteration)	3	
I detect new bad tastes in my mouth	4	
I did have changes in taste, but not anymore	5	**SKIP TO Q4a**
I do not perceive any change in taste perception	6	

Q3b.	**ONLY ASK THOSE WHO SELECTED 1-4 IN Q3a**<br>Thinking about the things that have changed your **sense of taste** (e.g. sweet, salt, sour, bitter, metallic taste), how much does it impact your daily life?<br><br>**CIRCLE ONE CODE ONLY**			
	Not at all	A little	Quite a bit	Very much
Taste	1	**2**	**3**	**4**

Q3c.	Thinking about the things that have changed your **sense of taste** (e.g. sweet, salt, sour, bitter, metallic taste), is your quality of life negatively affected because of these changes in taste?<br><br>**CIRCLE ONE CODE ONLY**			
	Not at all	A little	Quite a bit	Very much
Taste	1	**2**	**3**	**4**

Q4a.	How has your **sense of mouthfeel**, changed compared to before being positively tested for the corona virus, even if just a little?<br><br>**CIRCLE ALL THAT APPLY**	
I perceive mouthfeel different than before	1	**CONTINUE TO Q4b**
I do perceive a dry mouth	2	
I prefer other textures (e.g. thicker/thinner) than before	3	
I don't feel the tingling sensation of carbonated drinks anymore	4	
I did have changes in mouthfeel, but not anymore	5	**SKIP TO Q5a**
I do not perceive any change in mouthfeel perception	6	

Q4b.	**ONLY ASK THOSE WHO SELECTED 1-4 IN Q4a**<br>Thinking about the things that have changed your **sense of mouthfeel** (e.g. dry mouth feelings, temperature perception), how much does it impact your daily life?<br><br>**CIRCLE ONE CODE ONLY**			
	Not at all	A little	Quite a bit	Very much
Mouthfeel	1	**2**	**3**	**4**

Q4c.	Thinking about the things that have changed your **sense of mouthfeel** (e.g. dry mouth feelings, temperature perception)**,** is your quality of life negatively affected because of these changes in taste?<br><br>**CIRCLE ONE CODE ONLY**			
	Not at all	A little	Quite a bit	Very much
Mouthfeel	1	**2**	**3**	**4**

Q5a.	Do you have a change in preference on the **specific temperature at which food and drinks are served,** compared to before being positively tested for the corona virus**,** even if just a little?<br><br>**CIRCLE ALL THAT APPLY**	
Yes, I prefer frozen now	1	**CONTINUE TO Q5b**
Yes, I prefer chilled (fridge temperature) now	2	
Yes, I prefer ambient temperature now (around 20 degree Celsius)	3	
Yes, I prefer a little warmer now	4	
Yes, I prefer hot now	5	
My preferences was changed, but not anymore	6	
No, it remains the same	7	

Q5b.	**ONLY ASK THOSE WHO SELECTED 1-4 IN Q5a**<br>Thinking about the things that have changed your **specific temperature at which food and drinks are served,** how much does it impact your daily life?<br><br>**CIRCLE ONE CODE ONLY**			
	Not at all	A little	Quite a bit	Very much
Temperature	1	**2**	**3**	**4**

Q5c.	Thinking about the things that have changed your **specific temperature at which food and drinks are served,** is your quality of life negatively affected because of these changes in taste?<br><br>**CIRCLE ONE CODE ONLY**			
	Not at all	A little	Quite a bit	Very much
Temperature	1	**2**	**3**	**4**

- **IF RESPONDENT HAS SELECTED CODES 1-4 AT Q2a, ASK QUESTION Q6a.**
- **IF RESPONDENT HAS SELECTED CODE 5 AT Q2a AND IF RESPONDENT HAS SELECTED CODES 1-4 AT Q3a, ASK QUESTION Q7a.**
- **IF RESPONDENT HAS SELECTED CODE 5 AT Q2a AND Q3a, THE QUESTIONNAIRE IS COMPLETED.**

Smell

RESEARCHER: ASK QUESTION Q6a IF RESPONDENT HAS SELECTED CODES 1-4 AT Q2a (SMELL PERCEPTIONS HAVE CHANGED).

Q6a.	Thinking about your experiences of eating and drinking **since you have been tested positive for the corona virus**, to what level of severity did your smell perception change?<br><br>**CIRCLE ONE CODE ONLY**				
		Not at all	A little	Quite a bit	Very much
a.	Smell	1	**2**	**3**	**4**
			**CONTINUE TO Q6b**		

Q6b.	**ONLY ASK THOSE WHO SELECTED 2-4 IN Q6a**<br>How is the **course** of the smell changes since you have been tested positive for the corona virus?<br><br>**CIRCLE ONE CODE ONLY**						
		It decreases / I'm almost back at my old level.	It decreases a little	Stable	Increases a little	Increases	Fluctuating
	Course	1	2	3	4	5	6

Q6c.	Do you have continuous nasal congestion since you have been tested positive for the corona virus?<br><br>**CIRCLE ONE CODE ONLY**	
No	1	
At the start, but not anymore	2	
Yes	3	

Q6d. Generally, what do you do to cope with bad or distorted smells?<br><br>**CIRCLE ALL THAT APPLY**		
I don't have bad or distorted smells	1	
Nothing	2	
I choose colder foods (sandwiches, salads, fruits, and cheeses) that are less aromatic	3	
I open the windows and use the exhaust fan when cooking; open containers away from the face	4	
I eat other types of food that have smells I can bear	5	
Other, please specify:_______________	6	

Q6e. Generally, what do you do to cope with diminished smell?<br><br>**CIRCLE ALL THAT APPLY**		
I don't have a diminished smell	1	
Nothing	2	
I check more often if the gas is turned off	3	
I smell at products to check if I do smell something	4	
I ask other people to smell at possible rotten foods	5	
I pay more attention on personal hygiene (putting on clean clothes, extra deodorant)	6	
Other, please specify:_______________	7	

Q6f.	Can you please specify what, if any, odors you feel more sensitively than before?<br><br>**CIRCLE ALL THAT APPLY**	
		**Q12c**
Cleaning products	1	
Perfume	2	
Odor of the hospital room	3	
Fish	4	
Dairy (milk)	5	
Meat	6	
Acidic fruit	7	
Sweet fruits	8	
Savory	9	
Food cooking	10	
Other, please specify:______	11	
None	12	

- **IF RESPONDENT HAS SELECTED CODES 1-4 AT Q3a, ASK QUESTION Q7a.**
- **IF RESPONDENT HAS SELECTED CODE 5 AT Q3a, ASK QUESTION Q14**

TASTE

RESEARCHER: ASK QUESTION Q7a IF RESPONDENT HAS SELECTED CODES 1-4 AT Q3a (TASTE PERCEPTIONS HAVE CHANGED).

Q7a.	Thinking about your experiences of eating and drinking **since you have been tested positive for the corona virus**, to what level of severity did your taste perception change?<br><br>**CIRCLE ONE CODE ONLY**				
		Not at all	A little	Quite a bit	Very much
a.	Taste<br>(e.g. sweet, salty, sour, bitter, metallic etc.)	1	**2**	**3**	**4**
		**SKIP TO Q14**	**CONTINUE TO Q7b**		

Q7b.	**ONLY ASK THOSE WHO SELECTED 2-4 IN Q7a.**<br><br>How is the **course** of the taste changes since you have been tested positive for the corona virus?<br><br>**CIRCLE ONE CODE ONLY**						
		It decreases / I'm almost back at my old level.	It decreases a little	Stable	Increases a little	Increases	Fluctuating
	Course	1	2	3	4	5	6

Q7c. Generally, what do you do to cope with the **change in taste intensity**?

CIRCLE ALL THAT APPLY

Nothing	1	
Experiment with stronger flavors such as spices and marinades	2	
Avoid stronger flavors, like not adding spices or pepper	3	
Choose soft foods such as baked chicken, potatoes, paste and rice	4	
Consume foods with high moisture or water content	5	
Add sauces or gravy to moisten foods	6	
Other, please specify:...............................	7	

Q8a.	We would now like to ask you a bit more specifically about certain defined tastes. To what extent has the following **taste changed in intensity** for you?<br><br>**CIRCLE ONE CODE ONLY**				
	Much stronger	Slightly stronger	No change	Slightly weaker	Much weaker / cannot taste it at all
A change in things that normally would taste **salty**	1	2	3	4	5
	**CONTINUE TO Q8b**		**SKIP TO Q9a**	**CONTINUE TO Q8b**	

Q8b.	**ONLY ASK THOSE WHO SELECTED CODES 1, 2, 4 OR 5 at Q8a**<br>How much does the **change in salty taste** impact you?<br><br>**CIRCLE ONE CODE ONLY**			
	Not at all	A little	Quite a bit	Very much
**Salty taste**	1	**2**	**3**	**4**
	**SKIP TO Q9a**	**CONTINUE TO QUESTION Q8c**		

Q8c.	**ONLY ASK THOSE WHO SELECTED CODES 2-4 at Q8b**<br>What do you do to cope with this change in **salty taste**?<br><br>**CIRCLE ALL THAT APPLY**
Nothing	1
I limit salty foods and foods with added salt	2
I do not add salt during the cooking process or after meal preparation	3
I add fish sauce or soy sauce to the food	4
I add additional salt to the food	5
I add more chicken essence / chicken powder to the food	6
Other, please specify:______________	7

Q9a.	To what extent has the following taste changed in intensity for you?<br><br>**CIRCLE ONE CODE ONLY**					
		Much stronger	Slightly stronger	No change	Slightly weaker	Much weaker / cannot taste it at all
	A change in things that normally would taste **bitter**	1	2	3	4	5
		**CONTINUE TO Q9b**		**SKIP TO Q10a**	**CONTINUE TO Q9b**	

Q9b.	**ONLY ASK THOSE WHO SELECTED CODES 1, 2, 4 OR 5 at Q9a**<br>How much does the **change in bitter taste** impact you?<br><br>**CIRCLE ONE CODE ONLY**				
		Not at all	A little	Quite a bit	Very much
	**Bitter taste**	1	**2**	**3**	**4**
		**SKIP TO Q10a**	**CONTINUE TO QUESTION Q9c**		

Q9c.	**ONLY ASK THOSE WHO SELECTED CODES 2-4 at Q9b**<br>**ASK AS OPEN QUESTION**<br>What do you do to cope with this change in **bitter taste**?<br><br>**CIRCLE ALL THAT APPLY**	
Nothing	1	
I eat room-temperature or cold foods	2	
I limit intake of bitter products	3	
I try to make my food more sour (adding lime, lemon, vinegar or other)	4	
Other, please specify:______________	5	

Q10a.	To what extent has the following taste changed in intensity for you?<br><br>**CIRCLE ONE CODE ONLY**				
	Much stronger	Slightly stronger	No change	Slightly weaker	Much weaker / cannot taste it at all
A change in things that normally would taste **sweet.**	1	2	3	4	5
	**CONTINUE TO Q10b**		**SKIP TO Q11a**	**CONTINUE TO Q10b**	

Q10b.	**ONLY ASK THOSE WHO SELECTED CODES 1, 2, 4 OR 5 at Q10a**<br>How much does the **change in sweet taste** impact you?<br><br>**CIRCLE ONE CODE ONLY**			
	Not at all	A little	Quite a bit	Very much
**Sweet taste**	1	**2**	**3**	**4**
	**SKIP TO Q11a**	**CONTINUE TO QUESTION Q10c**		

Q10c. **ONLY ASK THOSE WHO SELECTED CODES 2-4 at Q10B.**<br>What do you do to cope with this change in sweet taste?<br><br>**CIRCLE ONE CODE ONLY**		
Nothing	1	
I limit sweet foods and foods with added sugar	2	
I add salt to sweet food	3	
I add more water to make the food less sweet	4	
I add sugar into the food	5	
I add sweeteners into the food such as strawberries, vanilla, etc.	6	
Other, please specify:______________	7	

Q11a.	To what extent has the following taste changed in intensity for you?<br><br>**CIRCLE ONE CODE ONLY**					
		Much stronger	Slightly stronger	No change	Slightly weaker	Much weaker / cannot taste it at all
	A change in things that normally would taste **sour.**	1	2	3	4	5
		**CONTINUE TO Q11b**		**SKIP TO Q12a**	**CONTINUE TO Q11b**	

Q11b.	**ONLY ASK THOSE WHO SELECTED CODES 1, 2, 4 OR 5 at Q11a**<br>How much does the **change in sour taste** impact you?<br><br>**CIRCLE ONE CODE ONLY**				
		Not at all	A little	Quite a bit	Very much
	**Sour taste**	1	**2**	**3**	**4**
		**SKIP TO Q12a**	**CONTINUE TO QUESTION Q11c**		

Q11c.	**ONLY ASK THOSE WHO SELECTED 2-4 AT VRAAG Q11b.**<br>What do you do to cope with this change in sour taste?<br><br>**CIRCLE ALL THAT APPLY**	
Nothing	1	
I limit sour foods such as citrus fruits.	2	
I add sugar into the food to make it less sour.	3	
I try to make the food more sour (adding lime, lemon, vinegar)	4	
Other, please specify:______________________________	5	

Q12a.	Do you experience any **metallic tastes** (e.g. imagine if you bite a key / smell your finger after you hold a key for a while) compared to before infection with the corona virus?<br><br>**CIRCLE ONE CODE ONLY**	
No	1	**SKIP TO Q13a**
Yes, I experience it without being presented with a food product	2	**CONTINUE TO Q12b**
Yes, I experience it only when being presented with a food product	3	

Q12b.	**ONLY ASK THOSE WHO SELECTED 2-3 IN Q12a**<br>Thinking about the things that make you experience any **metallic tastes,** how much does it impact your daily life?<br><br>**CIRCLE ONE CODE ONLY**			
	Not at all	A little	Quite a bit	Very much
Metallic tastes	1	**2**	**3**	**4**
	**SKIP TO Q13a**	**CONTINUE TO Q12c**		

Q12c. **ONLY ASK THOSE WHO SELECTED CODES 2-4 at Q12b**<br>What do you do to cope with this change in **metallic sensation**?<br><br>**CIRCLE ALL THAT APPLY**		
Nothing	1	
I limit red meat, liver, intestine, and other foods high in iron	2	
I experiment with other high-protein food choices such as egg, poultry, fish, beans and soy	3	
I use plastic utensils instead of metal	4	
I avoid consuming foods from metal cans and/or avoid cooking in an iron skillet	5	
Other, please specify:___________________	6	

Q13a.	Patients may experience a continuous taste in their mouth. Have you experienced a **continuous taste** in your mouth that you did not experience before your infection with the corona virus?<br><br>**CIRCLE ONE CODE ONLY**			
	Not at all	A little	Quite a bit	Very much
Continuous taste	1	**2**	**3**	**4**
		**CONTINUE TO Q13b**		

Q13b.	**ONLY ASK THOSE WHO SELECTED 2-4 IN Q13a**<br>Thinking about the things that make you experience any **continuous taste,** how much does it impact your daily life?<br><br>**CIRCLE ONE CODE ONLY**			
	Not at all	A little	Quite a bit	Very much
Continuous taste	1	2	3	4

13c.	Thinking about the things that make you experience any **continuous tastes,** which resembles this taste?<br><br>**CIRCLE ALL THAT APPLY**
1	Blood
2	Bitter
3	Something chemical
4	Something musty
5	Drugs
6	Metallic
7	Sweet
8	Salty
9	Sour
10	Other, please specify____

Q14.	**ONLY ASK THOSE WHO SELECTED 1-4 IN Q2a and/or Q3a**<br>Would you like more guidance for your changes in smell of taste? If yes, what kind of guidance?<br><br>**CIRCLE ALL THAT APPLY**	
No	1	
I would like a medicament (experimental) treatment	2	
I would like to receive more information about smell and taste changes	3	
I would like to receive help from a dietitian to advise me on adapting my food to my changes in smell and taste	4	
I would like to have my family receiving more information about my smell and taste changes	5	
I would like (more) peer support	6	
Other, please specify:__________________________	7	
